# Differences in Osteoimmunological Biomarkers Predictive of Psoriatic Arthritis among a Large Italian Cohort of Psoriatic Patients

**DOI:** 10.3390/ijms20225617

**Published:** 2019-11-10

**Authors:** Marco Diani, Silvia Perego, Veronica Sansoni, Lucrezia Bertino, Marta Gomarasca, Martina Faraldi, Paolo Daniele Maria Pigatto, Giovanni Damiani, Giuseppe Banfi, Gianfranco Altomare, Giovanni Lombardi

**Affiliations:** 1 Department of Dermatology and Venereology, IRCCS Istituto Ortopedico Galeazzi, 20161 Milan, Italy; marco.diani@unimi.it (M.D.); paolo.pigatto@unimi.it (P.D.M.P.); gianfranco.altomare@unimi.it (G.A.); 2 Laboratory of Experimental Biochemistry and Molecular Biology, IRCCS Istituto Ortopedico Galeazzi, 20161 Milan, Italy; silvia.perego@grupposandonato.it (S.P.); veronica.sansoni@grupposandonato.it (V.S.); marta.gomarasca@grupposandonato.it (M.G.); martina.faraldi@grupposandonato.it (M.F.); banfi.giuseppe@fondazionesanraffaele.it (G.B.); giovanni.lombardi@grupposandonato.it (G.L.); 3 Department of Clinical and Experimental Medicine, section of Dermatology, University of Messina, 98122 Messina, Italy; bertino.lucrezia@gmail.com; 4 Department of Biomedical, Surgical and Dental Sciences, University of Milan, 20122 Milano, Italy; 5 Department of Dermatology, Case Western Reserve University, Cleveland, OH 44106, USA; 6 Young Dermatologists Italian Network, Centro Studi GISED, 24121 Bergamo, Italy; 7 Vita-Salute San Raffaele University, 20132 Milan, Italy; 8 Department of Physiology and Pharmacology, Gdańsk University of Physical Education and Sport, 80336 Gdańsk, Poland

**Keywords:** psoriasis, psoriatic arthritis, osteoimmunological markers, bone resorption

## Abstract

(1) Background: In literature it is reported that 20–30% of psoriatic patients evolve to psoriatic arthritis over time. Currently, no specific biochemical markers can either predict progression to psoriatic arthritis or response to therapies. This study aimed to identify osteoimmunological markers applicable to clinical practice, giving a quantitative tool for evaluating pathological status and, eventually, to provide prognostic support in diagnosis. (2) Methods: Soluble (serum) bone and cartilage markers were quantified in 50 patients with only psoriasis, 50 psoriatic patients with psoriatic arthritis, and 20 healthy controls by means of multiplex and enzyme-linked immunoassays. (3) Results: Differences in the concentrations of matrix metalloproteases (MMPs), tissue inhibitors of metalloproteinases (TIMPs), receptor activator of nuclear factor kappa-B- ligand (RANK-L), procollagen type I N propeptide (PINP), C-terminal telopeptide of type I collagen (CTx-I), dickkopf-related protein 1 (DKK1), and sclerostin (SOST) distinguished healthy controls from psoriasis and psoriatic arthritis patients. We found that MMP2, MMP12, MMP13, TIMP2, and TIMP4 distinguished psoriasis from psoriatic arthritis patients undergoing a systemic treatment, with a good diagnostic accuracy (Area under the ROC Curve (AUC) > 0.7). Then, chitinase-3-like protein 1 (CHI3L1) and MMP10 distinguished psoriasis from psoriatic arthritis not undergoing systemic therapy and, in the presence of onychopathy, MMP8 levels were higher in psoriasis than in psoriatic arthritis. However, in these latter cases, the diagnostic accuracy of the identified biomarkers was low (0.5 < AUC < 0.7). (4) Conclusions. By highlighting never exploited differences, the wide osteoimmunological biomarkers panel provides a novel clue to the development of diagnostic paths in psoriasis and psoriasis-associated arthropathic disease.

## 1. Introduction

Adult psoriatic disease depicts a continuum encompassing disease progression from psoriasis (Ps) to psoriatic arthritis (PsA) [1]. In contrast to children, in adults, PsA manifests mainly after Ps and its musculoskeletal damages may be prevented with an early diagnosis and treatment [1,2]. Remarkably, 20–30% of Ps patients develop PsA but the non-clinically oriented instrumental screening of psoriatic patients is neither routinely adopted nor recommended due to the connected costs [1]. Thus, both clinical signs, such as psoriatic onychopathy and non-specific inflammatory parameters, are considered to identify Ps patients with a putative higher risk of developing PsA [3]. This is also accompanied by other proposed tools, such as the psoriasis epidemiology screening tool (PEST) questionnaire, evaluating the risk of developing PsA [4]. However, epidemiological studies based on clinical health records comparing Ps and PsA cohorts displayed contrasting results about the possible risk factors implicated in the evolution from Ps to PsA [5].

A biochemical marker that clearly predicts PsA in a cohort of Ps patients is still elusive. A biomarker is defined as a measurable characteristic indicating a biological or pathophysiological process and can be used to identify the risk to develop a certain disease [6]. Several studies investigated the association of different biomarkers with PsA. This condition has been associated with higher levels of cartilage oligomeric matrix protein (COMP), osteoprotegerin (OPG), matrix metalloproteinase 3 (MMP3), and the ratio between C-propeptide of type II collagen (CIIP) and collagen fragment neoepitope Col2–3/4 (C2C) [7]. Other studies found higher levels of CXCL10 in PsA patients compared to Ps patients [8], interleukin (IL)-6 [9], Dikkopf-1 (DKK-1), and macrophage-colony stimulating factor (M-CSF) [10]. However, the clinical significance of these studies is somehow limited due to the restricted panel of biomarkers considered and, more importantly, the lack of consideration of the therapeutic regimens and comorbidities/clinical signs (e.g., nail involvement).

Based on this background, this study aimed to investigate the possible association between a wide panel of osteoimmunological biomarkers with Ps and PsA, and in the relative sub-cohorts in order to identify a possible laboratory tool that could support PsA diagnosis and early prediction.

## 2. Results

### 2.1. Clinical Features of the Study Cohorts

Table 1 summarizes the study population demographics and clinical features.

In both Ps and PsA groups, a prevalence of male subjects, 66% and 78%, respectively, was observed despite no gender prevalence being reported. Median ages were 48 (19–82) years in Ps, 51(28–79) years in PsA, and 48 (29–67) years in controls. The body mass index (BMI), in the three groups, was 25 (23–29), 26 (23–28), and 24 (22–25) kg/m^2^, respectively.

The Psoriasis Area Severity Score (PASI) was higher in Ps (6 (5–12)) than in PsA (4 (1–7)). 

Onychopathic signs were present in 38% of Ps subjects and 48% of PsA subjects, according to the literature [11,12], PsA occurred after the diagnosis of Ps in 88% of patients. At recruitment, patients undergoing systemic therapy (ST) were 38% Ps and 34% PsA patients, respectively. Among these, 28 patients were treated with methotrexate (MTX, 13 Ps and 15 PsA), 6 with cyclosporine (CsA, 5 Ps and 1 PsA), 1 with acitretin (Ps), and 1 with cortisone (PsA).

### 2.2. Biochemical Characterization of the Study Cohorts

When considered in their entirety, without any subgrouping for treatment status, no significant differences were observed for any of the tested markers between Ps and PsA. Conversely, compared to the control group (CTRL), both Ps and PsA groups differed for most of the analyzed molecules, except for MMP3, MMP7, MMP12, tissue inhibitors of metalloproteinase (TIMP)-1, TIMP-2, OPG, C-telopeptide of type II collagen (CTx-II), and chitinase-3-like protein 1 (CHI3L1) (Table 2).

Furthermore, statistically significant correlations were found between markers’ concentration and duration of both Ps and PsA: MMP2, MMP12, MMP13, TIMP1, TIMP2, TIMP3, sclerostin (SOST), and CHI3L1 in Ps (positive correlation) and with MMP10 and TIMP2 in PsA (negative correlation). Moreover, in Ps group, MMP8, MMP10, and CTx-I positively correlated with PASI score, while TIMP4 was negatively correlated (Table 3).

### 2.3. Effect of Systemic Treatments

The Ps and PsA cohorts were further divided based on the therapy regimen (subjects undergone to systemic treatments (ST) and not systemically treated (NST)).

**Figure 1:**
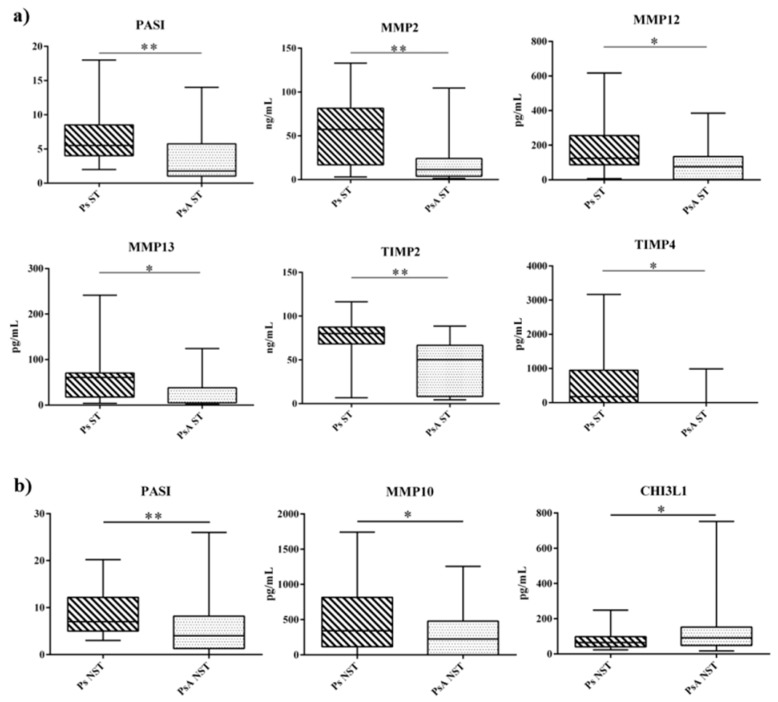
(**a**) Changes in PASI and serum profile of osteoimmunological markers in Ps ST group (hashed box) and PsA ST group (gray box). (**b**) Changes in PASI and serum profile of osteoimmunological markers in Ps NST group (hashed box) and PsA NST group (gray box). The box and whiskers plot identify, respectively, the value of the median (intermediate line), the 25th and 75th percentile (box), and the minimum and maximum value (whiskers). Asterisks indicate significant intergroup differences (* *p* < 0.05, ** *p* < 0.01). CHI3L1: Chitinase-3-like protein 1, MMP: Matrix metalloproteinases, NST: not systemically treated, PASI: Psoriasis area severity index, Ps: Psoriasis, PsA: Psoriatic arthritis, ST: systemically treated, TIMP: Tissue inhibitor of metalloproteinases.

When Ps and PsA subjects ST (*n* = 19 and *n* = 17, respectively) were compared, MMP2 (57.47 vs. 11.50 ng/mL, *p* = 0.006), MMP12 (124.10 vs. 76.43 pg/mL, *p* = 0.013), MMP13 (62.48 vs. 4.90 pg/mL, *p* = 0.029), TIMP2 (80.00 vs. 50.34 ng/mL, *p* = 0.001), and TIMP4 (177.7 vs. 1.7 pg/mL, *p* = 0.012) were higher in Ps to PsA (Figure 1A). As expected, PASI score was higher in Ps than in PsA patients (5.5 vs. 1.8, *p* = 0.004).

The Relative Operating Characteristic (ROC) analysis shows that the area under the ROC curve (AUC) for both the single markers (MMP2: 0.768, MMP12: 0.743, TIMP2: 0.811, TIMP4: 0.724) and their combination (0.755 to 0.845) display a moderately accurate diagnostic potential in discriminating Ps from PsA ST patients. Noteworthy, the combination of all these markers gave the highest diagnostic accuracy (AUC = 0.845) (Figure S1).

By focusing on the NST group, Ps NST (*n* = 31) and PsA NST (*n* = 33) significantly differed for MMP10 (340.9 vs. 224.9 pg/mL, *p* = 0.031), and CHI3L1 (65.21 vs. 91.54 pg/mL, *p* = 0.042) (Figure 1B), with the PASI score being always higher in Ps than in PsA (7 vs. 4, *p* = 0.002).

In Ps NST, MMP10 positively correlated with CHI3L1 (r = 0.446, *p* = 0.011), and negatively with PsA duration (r = −0.32, *p* = 0.024).

**Figure 2:**
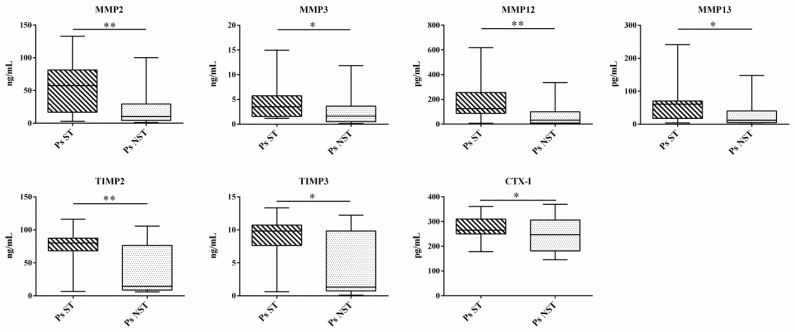
Changes in the serum profile of osteoimmunological markers in Ps ST group (hashed box) and Ps NST group (grey box). The box and whiskers plot identifies, respectively, the value of the median (intermediate line), the 25th and 75th percentile (box), and the minimum and maximum value (whiskers). Asterisks indicate significant intergroup differences (* *p* < 0.05, ** *p* < 0.01). CTx-I: C-terminal cross-linked telopetide of type I collagen, MMP: Matrix metalloproteinases, NST: not systemically treated, Ps: Psoriasis, PsA: Psoriatic arthritis, ST: systemically treated, TIMP: Tissue inhibitor of metalloproteinases.

From the ROC analysis emerges the AUC for these markers and their combinations are below 0.700 (Figure S2). Compared to Ps NST (*n* = 31), Ps ST (*n* = 19) had significantly increased serum levels of MMP2 (57.47 vs. 10.37 ng/mL, *p* = 0.007), MMP3 (3.52 vs. 1.65 ng/mL, *p* = 0.026), MMP12 (124.1 vs. 31.84 pg/mL, *p* = 0.002), MMP13 (62.48 vs. 12.21 pg/mL, *p* = 0.013), TIMP2 (80.00 vs. 14.34 ng/mL, *p* = 0.003), TIMP3 (9.83 vs. 1.33 ng/mL, *p* = 0.022), and CTx-I (265.2 vs. 246.8 pg/mL, *p* = 0.01) (Figure 2).

Interestingly, no osteoimmunological biomarkers were statistically different in the comparison PsA ST (*n* = 17) vs. PsA NST (*n* = 33).

### 2.4. Onychopathy Biochemical Signature

**Figure 3:**
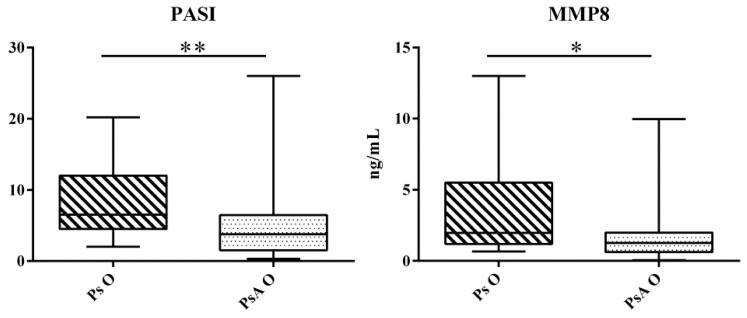
Changes in PASI and serum profile of MMP8 in Ps O group (hashed box) and PsA O group (dotted box). The box and whiskers plot identifies, respectively, the value of the median (intermediate line), the 25^th^ and 75^th^ percentile (box), and the minimum and maximum value (whiskers). Asterisks indicate significant intergroup differences (* *p* < 0.05, ** *p* < 0.01). MMP: Matrix metalloproteinasis, PASI: Psoriasis area severity index, Ps O: Psoriatic onychopathy, PsA O: Psoriatic arthritis with onychopathy.

Ps patients with onychopathy (Ps O) had higher PASI (6.5 vs. 3.7 *p* = 0.009) and higher circulating MMP8 (1.96 vs. 1.26 ng/mL, *p* = 0.028) compared to onychopathic PsA patients (PsA O) (Figure 3). ROC analysis gave an AUC below 0.700, for MMP8 (Figure S3).

Non-onychopathic Ps compared to PsA counterparts achieved significance only for PASI (6.6 vs. 1.7 *p* = 0.002). 

## 3. Discussion

The present study investigated two different possible sets of osteoimmunological biomarkers to screen for PsA among Ps. This is of particular interest because Ps patients, especially those with a mild cutaneous involvement, having a subclinical PsA are hardly identifiable using standard measures. The consequent delayed diagnosis of PsA results in a more severe impact on the joint status. Imaging techniques have a high diagnostic potential, but they are intrinsically limited in terms of predictability. Contrarily, since biochemical markers expression anticipates the micro/macro-structural changes, they have a predictive potential. However, markers discriminating between Ps and PsA have not been identified, although advances have been gained in the biochemical differential characterization between RA and seronegative arthropathies [13].

Previous findings gave controversy results, eventually due to the high within-cohort heterogeneity. Indeed, in our study, when the entire cohorts are considered, the main differences were between Ps and PsA and healthy controls. Similarly, previous studies failed in determining any difference between Ps and PsA in sRANKL, COMP, and OPG concentrations. A possible explanation of differences found comparing Ps and PsA patients with their healthy counterparts is that, actually, some Ps patients that are asymptomatic for PsA are, instead, suffering from subclinical cartilage and bone changing [14]. In particular, the pathological changes in cartilage and bone can be associated with enthesitis, dactylitis, nail dystrophy, and new bone apposition. Importantly, aberrant bone formation characterizes and clearly differentiates PsA from rheumatoid arthritis (RA) and other inflammatory arthritis where resorptive phenomena prevail [15,16]. On the other hand, the inclusion of patients under different treatment regimens and/or with different clinical features within the same cohorts makes the comparison less fine.

A first set of biomarkers, composed matrix metalloproteinases, and their tissue inhibitors comprises of MMP2, MMP12, MMP13, TIMP2, and TIMP4 whose circulating levels are higher in Ps undergoing systemic therapy compared to the parallel PsA cohort. Moreover, the ROC analysis of both single and differently combined markers (even more, the combination of all five) gave a good diagnostic accuracy. Consequently, this panel is particularly promising because of the wide use of MTX in both Ps and PsA. The effects of systemic treatments on the osteoimmune function is clearly depicted, in our study, by the decrease in the circulating levels of most of the markers here considered. 

A second set of biomarkers seems to be useful in differentiating between Ps and PsA that have never undergone systemic treatments (NST). Given their naïve condition, the comparison of Ps NST and PsA NST patients could be considered as the most useful. This set comprises of MMP10 and CHI3L1 whose levels are, moreover, reciprocally correlated. CHI3L1 is an inactive chitinase due to the lack of the catalytic domain whose physiological functions have not been fully clarified, although it may play a role in inflammation [17,18,19]. Biological activities of CHI3L1 include regulation of cell proliferation, adhesion, migration, and activation. Furthermore, CHI3L1 is produced by a variety of inflammatory cells, including neutrophils, monocytes/macrophages, and osteoclasts [20], and its induction has been reported in patients suffering from many diseases, including several autoimmune disorders [21]. In addition, elevated plasma levels of CHI3L1 have also been found in RA [22]. According to previous findings, Ps patients experience higher CHI3L1 circulating levels than healthy subjects [23]. Our result seems to indicate that this protein could be a new inflammatory biomarker associated with PsA. 

Nail psoriasis is, among others, a risk factor for the development of PsA, especially within the distal interphalangeal joints, as suggested by McGonagle and colleagues [24,25]. Remarkably, the enthesistis of the extensor tendon of the finger in the distal interphalangeal joint of the hand may clinically manifest with psoriatic onychopathy [26]. Although it is reported in literature that psoriatic onychopathy appears in 50% of Ps and in 93% of PsA patients [27], PsA is diagnosed after Ps development in 88% of adult patients. Several epidemiological studies associated psoriatic onychopathy with Ps duration, Ps early onset, high PASI, and concurrent PsA [28]. Systemic therapies, both biologics and non-biological, are regarded as effective and as a possible secondary treatment step in case of lack/loss of response to topical therapies in patients with high PASI or high DLQI (the dermatology life quality index) or PsA [29]. MTX and CsA can improve the nail lesions, with MTX being more effective in treating nail matrix changes, while CsA is more effective in improving nail bed scores [30]. 

In those subjects presenting onychopathy signs, MMP8 concentrations were higher in Ps than in PsA. Being a collagenase, MMP8 preferentially cleaves the interstitial type I collagen and is the first collagenase that appears during dermal wound-healing [31,32]. MMP8 is expressed by keratinocytes, fibroblasts, and granulocytes, at the level of the cutaneous lesions, and by synovial cells. In this latter case it was hypothesized that MMP8 solves a function in osteo-articular remodeling [33]. It is known that MMP-expressing cells are found in high amounts in the flogotic joint [34]. Therefore, it could be hypothesized that the circulating levels of MMP8 in PsA patients with onychopathy, compared to their Ps counterparts, were low because of its release into the close joint structures rather than into the blood stream. In fact, in PsA patients, as in those with RA, the levels of MMPs are elevated within the joint, which is a closed environment [35]. On the contrary, in Ps subjects without any joint involvement, MMP8 is released directly into the bloodstream, since expressed at the skin level which is, instead, an open system. Although possible, this speculation needs to be demonstrated.

The cross-sectional nature of this study and the somehow limited sample size (pilot study) represent the main limitations, although this is compensated by the great homogeneity of the selected cohorts that limits the number of variables potentially affecting the circulating levels of the analyzed biomarkers. Remarkably, due to the exiguity of the sample, it was not possible to evaluate the influences of drugs and severity on the biomarker’s levels. Future studies are mandatory to assess the osteoimmunological biomarker specificity for PsA and further compare the present findings with other autoimmune diseases involving joints, such as RA or gout.

## 4. Methods 

### 4.1. Ethical Committee

The clinical trial was approved by the ethical committee (BioArp, Ospedale San Raffaele, Milano, Italia), and registered at clinicaltrials.gov (NCT03455166). The study was carried out following the rules of the Declaration of Helsinki of 1975, revised in 2008 (https://www.wma.net/what-we-do/medical-ethics/declaration-of-helsinki/).

A written consent to the use of clinical data was obtained from all patients after being informed about the study procedures, their benefits, and eventual risks.

### 4.2. Patients Selection and Study Design

Patients with a diagnosis of plaque psoriasis (Ps, *n* = 50) and patients with Ps and PsA (PsA, *n* = 50) were enrolled at the department of dermatology and venereology of the IRCCS Istituto Ortopedico Galeazzi (Milan, Italy), starting from June 2015 until April 2017, during routine clinical activities. Inclusion criteria for Ps patients were plaque psoriasis (no erythrodermic, inverse, or guttate forms) for more than six months, neither current nor previous treatment with biological drugs, and no joint involvement.

Inclusion criteria for PsA patients fulfilling classification for psoriatic arthritis (CASPAR) criteria [36] were active peripheral (disease activity index for psoriatic arthritis (DAPSA) [37] ≥5 points) and axial arthropathy (Bath ankylosing spondylitis disease activity index (BASDAI) [38] ≥4 points), as well as enthesitis (Leeds enthesitis index (LEI) [39] >0 points), and dactylitis (dactylitis severity score (DSS) [40] >2 points) with cutaneous involvement (PASI >3), neither current treatment with biological drugs nor biological treatment. For PsA, we included a new diagnosis of PsA in order, but also of patients without an experience with biologics or patients unresponsive to biological treatments willing to start a new systemic treatment, or, if they experienced biologics, they stopped them in favor of a systemic conventional treatment for a minimum of 4 months before entering in this study. Remarkably, patients should have suspended topical therapy for at least 4 weeks.

Psoriatic onychopathy was included only if nail psoriasis severity index (NAPSI) [41] ≥ 3 points.

Exclusion criteria, common to the two groups, included pregnancy, current or previous malignancies, other acute or chronic inflammatory diseases, infectious diseases (human immunodeficiency virus (HIV), hepatitis B and C [42], tuberculosis), other rheumatologic diseases, primary bone metabolic diseases, recent bone fractures (within 6 months), anxiety, psychosis, or depressive disorders, supplementations, or particular diets (included fasting) [43,44,45]. 

### 4.3. Dermatology and Rheumatology Assessment of Patients

During the outpatient visit, Ps history, disease onset and evolution were evaluated. The most prevalent type of Ps was determined, along with the body sites involved and the presence of the onychopathic trait. A psoriasis area and severity index (PASI) score was assigned by two independent board-certified dermatologists. 

PsA diagnosis was formulated by two independent board certified rheumatologists prior to the dermatological examination, based on anamnestic and clinical evaluations of the patient’s inflammatory and imaging indexes. In particular, based on CASPAR criteria [36] and ultrasound (US) or nuclear magnetic resonance imaging (NMRI), the specialist evaluated the presence of Ps, signs and symptoms of joint inflammation (i.e., dactylitis, enthesitis), and reduced joint mobility. 

Finally, laboratory analyses completed the diagnostic path by investigating markers useful in the differential diagnosis with other forms of inflammatory arthritis (e.g., rheumatoid factor, anti-CCP, inflammatory indices).

### 4.4. Blood Sampling and Biochemical Determinations

Blood samples were collected by standard venipuncture of the antecubital vein in SST II Advance Vacutainer^®^ (Becton, Dickinson and Co., Franklin Lakes, NJ, USA). Blood was immediately centrifuged at 1300× *g* (10 min, 15 °C) and serum was aliquoted and stored at −80 °C until assayed. Serum samples from 20 healthy Caucasian age- and sex-matched subjects (10 males and 10 females, Cliniscience, Nanterre, FR, EU) were used as a control (CTRL).

Serum matrix metalloproteinases (MMP1, MMP2, MMP3, MMP7, MMP8, MMP9, MMP10, MMP12, MMP13) and their tissue inhibitors (TIMP1, TIMP2, TIMP3, TIMP4) were quantified using a multiplex assay on a Bio-Plex^®^ Multiplex System (Bio-Rad Laboratories Inc., Hercules, CA, USA).

Osteoimmune markers, OPG, and RANKL, concentrations were measured in serum using monoclonal antibody-based immunoassays (ELISA) (BioVendor-Laboratorni Medicina A.S., Brno, CZ, EU). A marker of bone resorption, CTx-I, and a marker of bone formation, PINP, were measured in serum by monoclonal antibody-based immunoassays (Cloud-Clone Corp^©^, Houston, TX, USA). Serum markers of cartilage degradation and inflammation, CTx-II and CHI3L1, also known as YKL-40, were measured by a competitive enzyme immunoassay (Cloud-Clone Corp^©^) and a sandwich enzyme immunoassay (BioVendor-Laboratorni Medicina A.S.), respectively. Inhibitors of the Wnt signaling pathway, DKK1, and SOST, were assayed in serum by a solid-phase ELISA (Quantikine^®^ R&D Systems Inc., Minneapolis, MN, USA).

The lower limits of detection (LOD) were 0.03 pmol/L for OPG, 0.4 pmol/L for RANKL, 44.3 pg/mL for CTx-I, 12.4 pg/mL for PINP, 52.3 pg/mL for CTx-II, 5.0 pg/mL for CHI3L1, 4.20 pg/mL for DKK1, and 1.78 pg/mL for SOST. Intra-assay (CV_w_) and inter-assay (CV_b_) variations were: 2.5–4.9% and 1.7–9.0% for OPG, 7.25–11.51% and 11.21–12.77% for RANKL, <10% and <12% for CTx-I, PINP, CTx-II, CHI3L1, 4.2% and 7.6% for DKK1, 2.1% and 10.8% for SOST.

Strict warnings were applied during the pre-analytical phase (i.e., collection, handling, and storage) in order to limit variability in the final analytical output [12,13].

### 4.5. Statistical Analysis

Shapiro–Wilk’s normality test was performed on data from the entire cohort. Since the non-parametrical distribution of the values, quantitative parameters are expressed as the median and the interquartile range in the descriptive analysis. The within-group comparisons were performed using Mann–Whitney U-test. Kruskall–Wallis test was used for multiple comparison. Spearman’s rank correlation test was applied to evaluate correlations that were considered significant when r ≥ 0.25. 

Diagnostic accuracy of those markers displaying a statistically significant difference in the subgroup’s comparison was determined by the ROC (receiver operating characteristic) curves analysis. The AUC (area under curve)-based accuracy was classified according to Swets JA [14].

The significance was set at *p* < 0.05. Analyses were performed using SPSS software (IBM, Armonk, NY, USA).

## 5. Conclusions

This study, comparing a large panel of osteoimmunological biomarkers, highlighted profound differences between Ps, PsA, and healthy controls. These phenotypic differences, which are attenuated in the comparison between Ps and PsA, reinforces the thesis according to which these two diseases belong to the same pathological spectrum. However, we also identified important differences in the expression of specific tissue-remodeling associated enzymatic activities in selected sub-cohorts of Ps and PsA subjects that seem to be dependent upon the treatment (systemic vs. topic) and the presence nail involvement (onychopathy).

## Figures and Tables

**Table 1:** Clinical features of patients at the time of recruitment.

Variables	Total Cases (*n* = 100)	Ps Group (*n* = 50)	PsA Group (*n* = 50)	CTRL Group (*n* = 20)
Age median (IQR), years	49 (19–82)	48 (19–82)	48 (28–79)	48 (29–67)
Female, *n* (%)	28 (28)	17 (34)	11 (22)	10 (50)
Male, *n* (%)	72 (72)	33 (66)	39 (78)	10 (50)
BMI median (IQR), kg/m^2^	25 (23–28)	25 (23–29)	26 (23–28)	24 (22–25)
Ps duration median (IQR), months	195 (83–319)	200 (67–347)	195 (110–317)	-
Eruptive/stable Ps, (*n*)	-	39/11		-
PsA duration median (IQR), months	-	-	25 (4–110)	-
PASI median (IQR)	5 (2–8)	6 (5–12)	4 (1–7)	-
Onychopathy (*n*)	43	19	24	-
Non systemic therapy (*n*)	64	31	33	-
Systemic therapy (*n*)	36	19	17	-
Acitretin (*n*) (dose)	1 (20 mg/die)	1 (20 mg/die)	0	-
CsA (*n*) (dose)	6 (237.5 (221.3–257.5) mg/die)	5 (250 (220–260) mg/die)	1 (225 mg/die)	-
MTX (*n*) (dose)	28 (15 (15–17.5) mg/week)	13 (15 (15–17.5) mg/die)	15 (15 (15–16.25) mg/die)	-
Systemic prednisone (*n*) (dose)	1 (10 mg/die)	-	1 (10 mg/die)	-

BMI: Body mass index, CsA: Cyclosporin, CTRL: Controls, IQR: Interquartile range, MTX: Methotrexate, *n*: number, Ps: Psoriasis, PsA: Psoriatic arthritis.

**Table 2:** Concentration of serum osteoimmunological markers measured in the study cohorts.

Markers	CTRL Group (*n* = 20)	Ps Group (*n* = 50)	PsA Group (*n* = 50)	CTRL vs. Ps *p*-Value	CTRL vs. PsA *p*-Value
MMP1 (pg/mL)	35.00 (35.00–35.00)	471.20 (159.10–1033.00)	608.00 (35.00–1266.00)	*p* < 0.001 ⇑	*p* = 0.001 ⇑
MMP2 (ng/mL)	91.70 (74.58–102.26)	21.18 (7.8–76.60)	16.52 (4.75–78.11)	*p* < 0.001 ⇓	*p* < 0.001 ⇓
MMP3 (ng/mL)	6.27 (3.10–11.67)	2.39 (1.14–4.93)	2.29 (0.74–4.44)	n.s	n.s
MMP7 (ng/mL)	0.86 (0.47–1.16)	0.42 (0.22–1.08)	0.38 (0.19–1.31)	n.s	n.s
MMP8 (ng/mL)	0.61 (0.51–0.71)	1.35 (0.83–3.01)	1.32 (0.71–3.15)	*p* = 0.001 ⇑	*p* = 0.009 ⇑
MMP9 (ng/mL)	0.83 (0.61–1.25)	8.22 (3.31–11.72)	6.59 (3.35–11.63)	*p* < 0.001 ⇑	*p* < 0.001 ⇑
MMP10 (pg/mL)	1.60 (1.60–1.60)	317 (1.60–824.30)	257.47 (1.60–541.60)	*p* < 0.001 ⇑	*p* < 0.001 ⇑
MMP12 (pg/mL)	1.00 (1.00–444.20)	86.63 (11.76–165.40)	76.99 (7.65–144.10)	n.s	n.s
MMP13 (pg/mL)	4.90 (4.90–4.90)	24.8 5(4.90–63.21)	4.90 (4.90–49.19)	*p* = 0.004 ⇑	n.s
TIMP1 (ng/mL)	78.74 (66.65–109.45)	101.19 (17.17–114.69)	85.53 (17.71–113.35)	n.s	n.s
TIMP2 (ng/mL)	90.71 (72.38–105.16)	70.77 (10.09–83.73)	59.83 (8.91–76.81)	*p* = 0.011 ⇓	*p* < 0.001 ⇓
TIMP3 (ng/mL)	0.09 (0.09–1.66)	8.79 (0.76–10.73)	6.98 (0.61–9.17)	*p* < 0.001 ⇑	*p* < 0.001 ⇑
TIMP4 (pg/mL)	1072.75 (701.90–1934.00)	27.11 (1.70–320.90)	1.70 (1.70–175.20)	*p* < 0.001 ⇓	*p* < 0.001 ⇓
OPG (pmol/L)	6.46 (3.83–8.51)	5.58 (4.23–6.99)	5.67 (4.51–6.95)	n.s	n.s
RANKL (pmol/L)	393.95 (295.50–943.00)	148.20 (81.75–293.20)	165.40 (86.79–235.20)	*p* < 0.001 ⇓	*p* < 0.001 ⇓
PINP (ng/mL)	46.17 (33.55–62.88)	5.94 (5.01–7.40)	7.08 (4.87–8.22)	*p* < 0.001 ⇓	*p* < 0.001 ⇓
CTx-I (ng/mL)	1.70 (1.38–2.14)	0.55 (0.44–0.61)	0.51 (0.40–0.62)	*p* < 0.001 ⇓	*p* < 0.001 ⇓
CTx-II (ng/mL)	0.25 (0.19–0.29)	0.26 (0.20–0.31)	0.26 (0.20–0.30)	n.s	n.s
DKK1 (ng/mL)	0.27 (0.23–0.37)	2.79 (2.30–3.74)	2.80 (2.02–3.53)	*p* < 0.001 ⇑	*p* < 0.001 ⇑
SOST (pg/mL)	55.17 (35.37–97.10)	147.95 (111.20–186.70)	154.20 (126.90–196.60)	*p* < 0.001 ⇑	*p* < 0.001 ⇑
CHI3L1 (pg/mL)	70.19 (31.87–118.80)	65.98 (47.81–107.00)	83.11 (47.33–114.90)	n.s	n.s

Measures are expressed as median (IQR). CHI3L1: chitinase-3-like protein 1, CTRL: controls, CTx-I: C-terminal cross-linked telopetide of type I collagen, CTx-II: C-terminal cross-linked telopeptides of type II collagen, DKK1: Dickkopf-related protein 1, IQR: Interquartile range, MMP: Matrix metalloproteinases, n.s.: Not significant, PINP: procollagen type I N-terminal propeptide, Ps: Psoriasis, PsA: Psoriatic arthritis, RANKL: receptor activator of NF-κB ligand, TIMP: tissue inhibitor of metalloproteinases, SOST: sclerostin.

**Table 3:** Correlation analysis between osteoimmunological biomarker concentrations and duration of disease or PASI score. Correlations reaching statistical significance are given in bold.

Markers	Ps Duration		PsA Duration		Ps PASI		PsA PASI	
	*p*	r	*p*	r	*p*	r	*p*	r
MMP1	0.486	−0.10	0.790	−0.04	0.216	0.18	0.647	0.07
MMP2	**0.001**	**0.46**	0.929	0.01	0.322	−0.14	0.205	−0.18
MMP3	0.549	0.09	0.458	0.11	0.459	−0.11	0.829	−0.03
MMP7	0.651	0.07	0.120	−0.23	0.316	0.14	0.746	−0.05
MMP8	0.177	0.19	0.098	−0.24	0.040	0.29	0.054	0.27
MMP9	0.630	0.07	0.306	−0.15	0.257	0.16	0.226	0.17
MMP10	0.876	0.02	**0.025**	**−0.32**	**0.004**	**0.40**	0.057	0.27
MMP12	**0.001**	**0.45**	0.418	−0.12	0.372	−0.13	0.313	−0.15
MMP13	**0.010**	**0.36**	0.392	−0.13	0.667	−0.06	0.781	−0.04
TIMP1	**0.016**	**0.34**	**0.049**	**−0.29**	0.935	0.01	0.361	−0.13
TIMP2	**0.037**	**0.30**	0.203	−0.19	0.871	0.02	0.372	−0.13
TIMP3	**0.042**	**0.29**	0.087	−0.25	0.946	0.01	0.709	−0.05
TIMP4	0.065	0.26	0.052	0.28	**0.041**	**−0.29**	0.497	−0.10
OPG	0.341	0.14	0.176	0.20	0.067	−0.26	0.448	−0.11
RANKL	0.335	−0.14	0.616	−0.07	0.450	−0.11	0.680	0.06
PINP	0.571	−0.08	0.638	−0.07	0.999	0.00	0.480	0.10
CTX-I	0.215	−0.18	0.341	0.14	**0.048**	**0.28**	0.440	0.11
CTX-II	0.398	0.12	0.956	−0.01	0.124	−0.22	0.161	−0.20
DKK1	0.233	0.17	0.684	−0.06	0.474	−0.10	0.985	0.00
SOST	**0.015**	**0.34**	0.343	−0.14	0.286	−0.15	0.421	−0.12
CHI3L1	**0.010**	**0.36**	0.384	0.13	0.284	−0.15	0.596	0.08

CHI3L1: Chitinase-3-like protein 1, CTx-I: C-terminal cross-linked telopetide of type I collagen, CTx-II: C-terminal cross-linked telopeptides of type II collagen, DKK1: Dickkopf-related protein 1, MMP: Matrix metalloproteinases, PASI: Psoriasis area severity index, *p*: *p* value, PINP: Procollagen type I N-terminal propeptide, Ps: Psoriasis, PsA: Psoriatic arthritis, RANKL: Receptor activator of NF-κB ligand, r: Pearson coefficient, TIMP: Tissue inhibitor of metalloproteinases, SOST: Sclerostin.

## Supplementary Materials

Supplementary Materials can be found at https://www.mdpi.com/1422-0067/20/22/5617/s1.
