# Supplementary material for: Differences in Osteoimmunological Biomarkers Predictive of Psoriatic Arthritis among a Large Italian Cohort of Psoriatic Patients
Source: Int J Mol Sci. 2019 Nov 10;20(22):5617. doi: 10.3390/ijms20225617 (PMC6888436; doi:10.3390/ijms20225617)

Supplementary material

Figure S1: Roc Curve Analyses of both single markers and their combinations in patients undergone to systemic therapy.

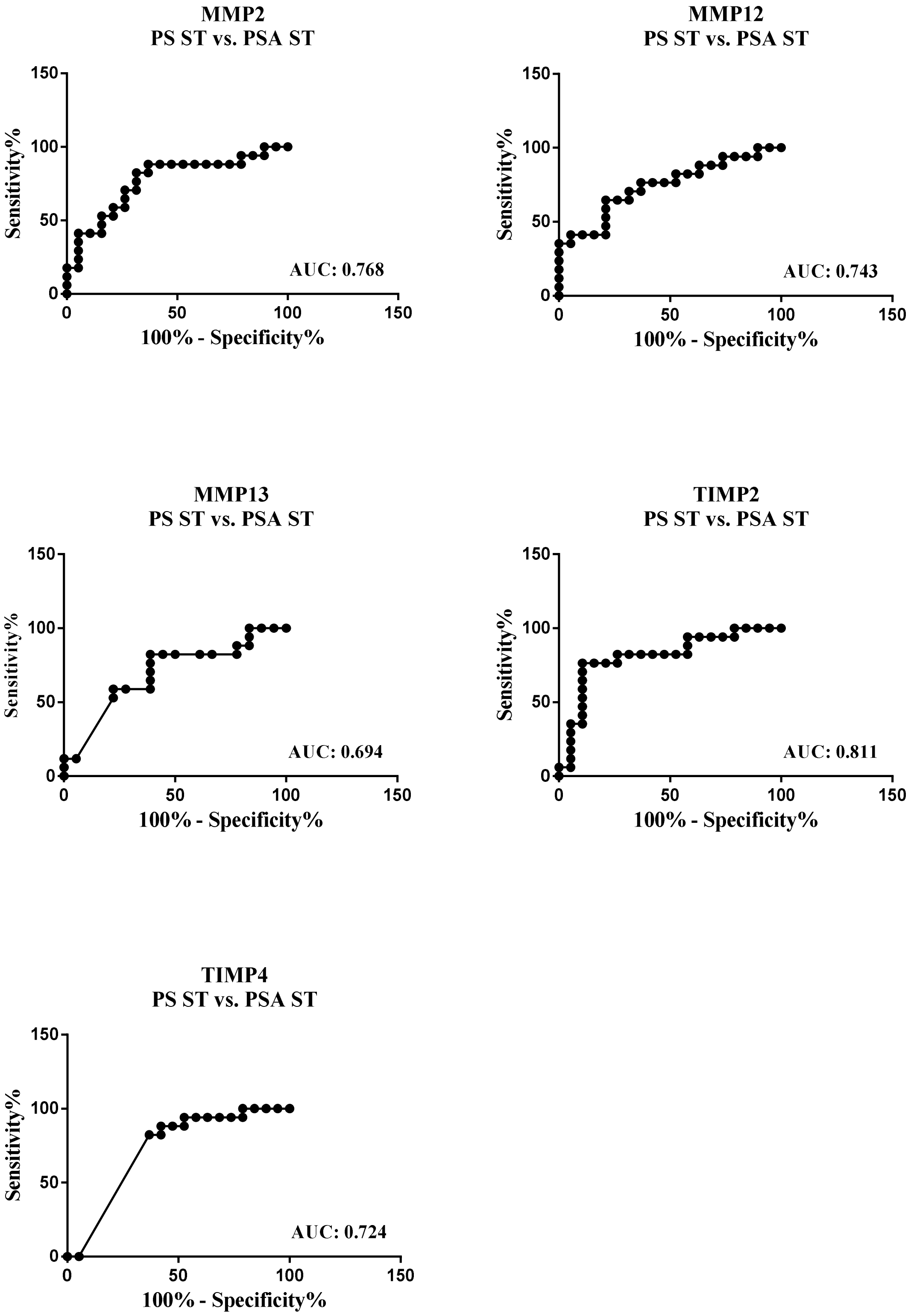

MMP2+MMP12
PS NST vs. PSA NST

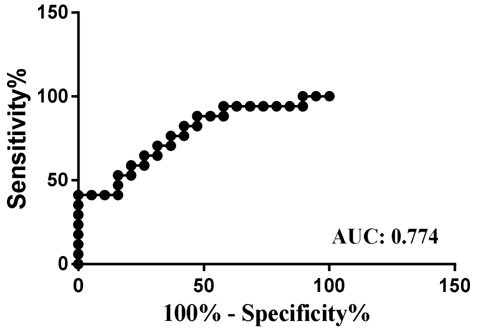

MMP2+MMP13
PS NST vs. PSA NST

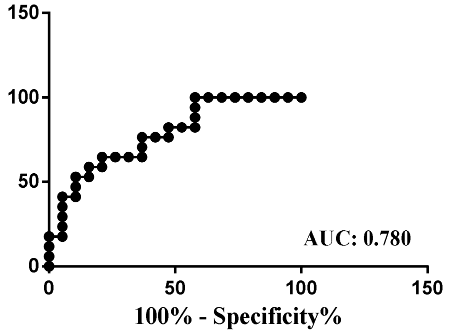

MMP2+TIMP2
PS NST vs. PSA NST

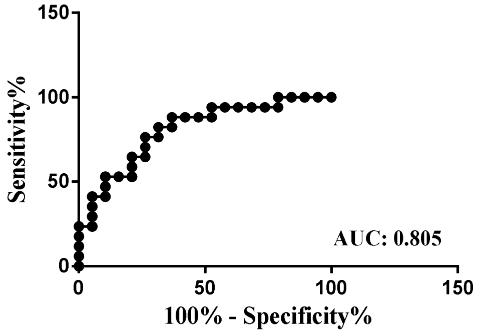

MMP2+TIMP4
PS NST vs. PSA NST

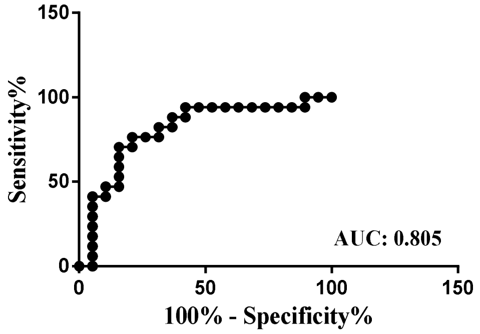

MMP12+MMP13
PS NST vs. PSA NST

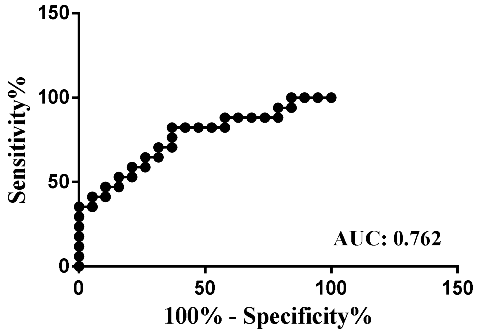

MMP12+TIMP2
PS NST vs. PSA NST

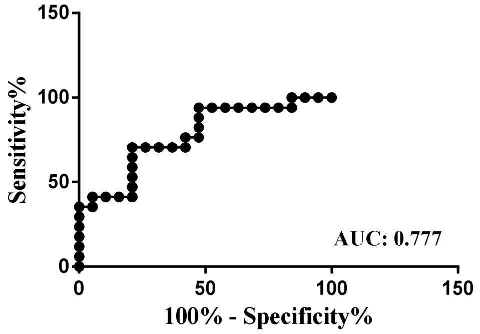

MMP12+TIMP4
PS NST vs. PSA NST

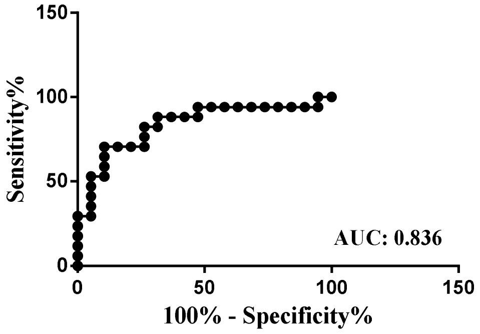

MMP13+TIMP2
PS NST vs. PSA NST

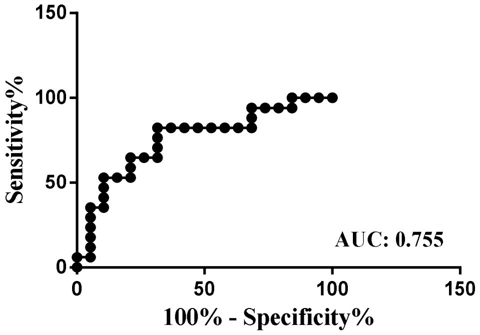

MMP13+TIMP4
PS NST vs. PSA NST

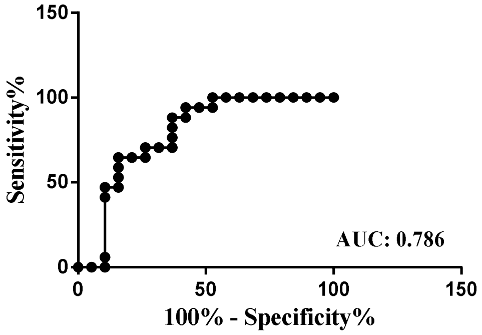

TIMP2+TIMP4
PS NST vs. PSA NST

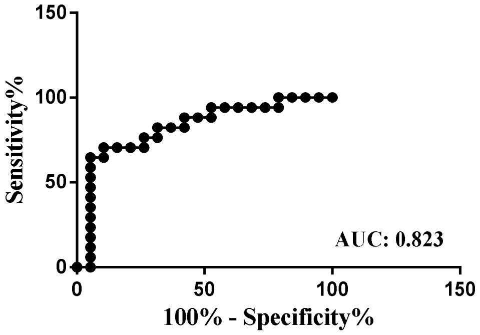

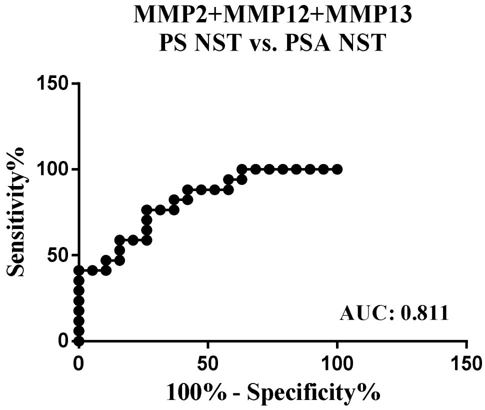
MMP2+MMP12+MMP13
PS NST vs. PSA NST
Sensitivity%
100% - Specificity%
AUC: 0.811
0
50
100
150

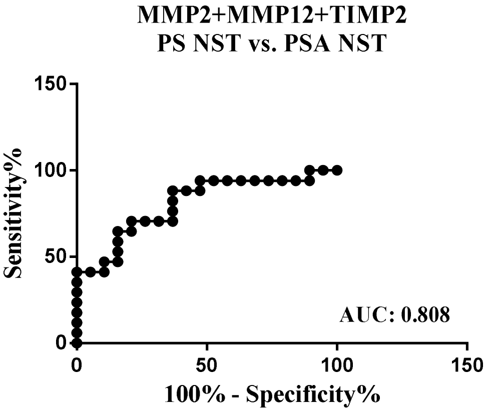
MMP2+MMP12+TIMP2
PS NST vs. PSA NST
Sensitivity%
100% - Specificity%
AUC: 0.808
0
50
100
150

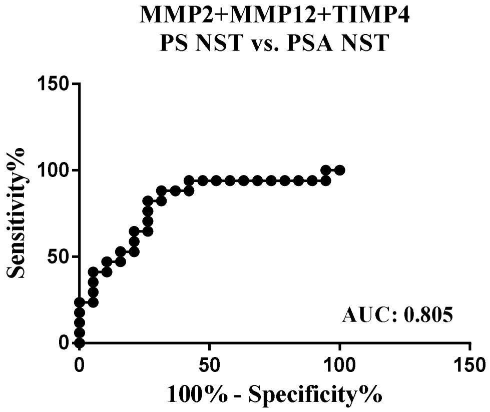
MMP2+MMP12+TIMP4
PS NST vs. PSA NST
Sensitivity%
100% - Specificity%
AUC: 0.805
0
50
100
150

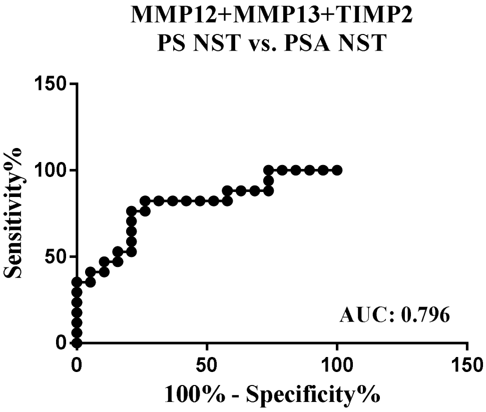
MMP12+MMP13+TIMP2
PS NST vs. PSA NST
Sensitivity%
100% - Specificity%
AUC: 0.796
0
50
100
150

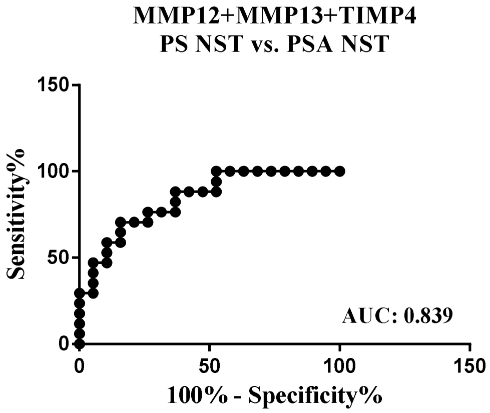
MMP12+MMP13+TIMP4
PS NST vs. PSA NST
Sensitivity%
100% - Specificity%
AUC: 0.839
0
50
100
150

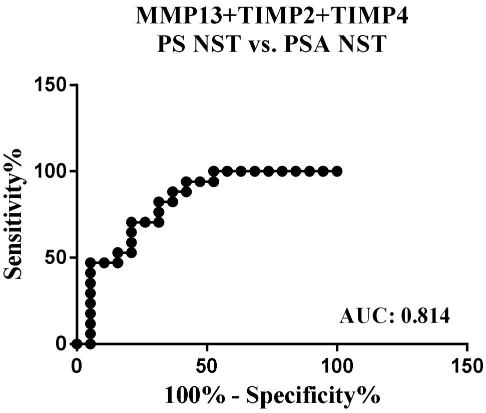
MMP13+TIMP2+TIMP4
PS NST vs. PSA NST
Sensitivity%
100% - Specificity%
AUC: 0.814
0
50
100
150

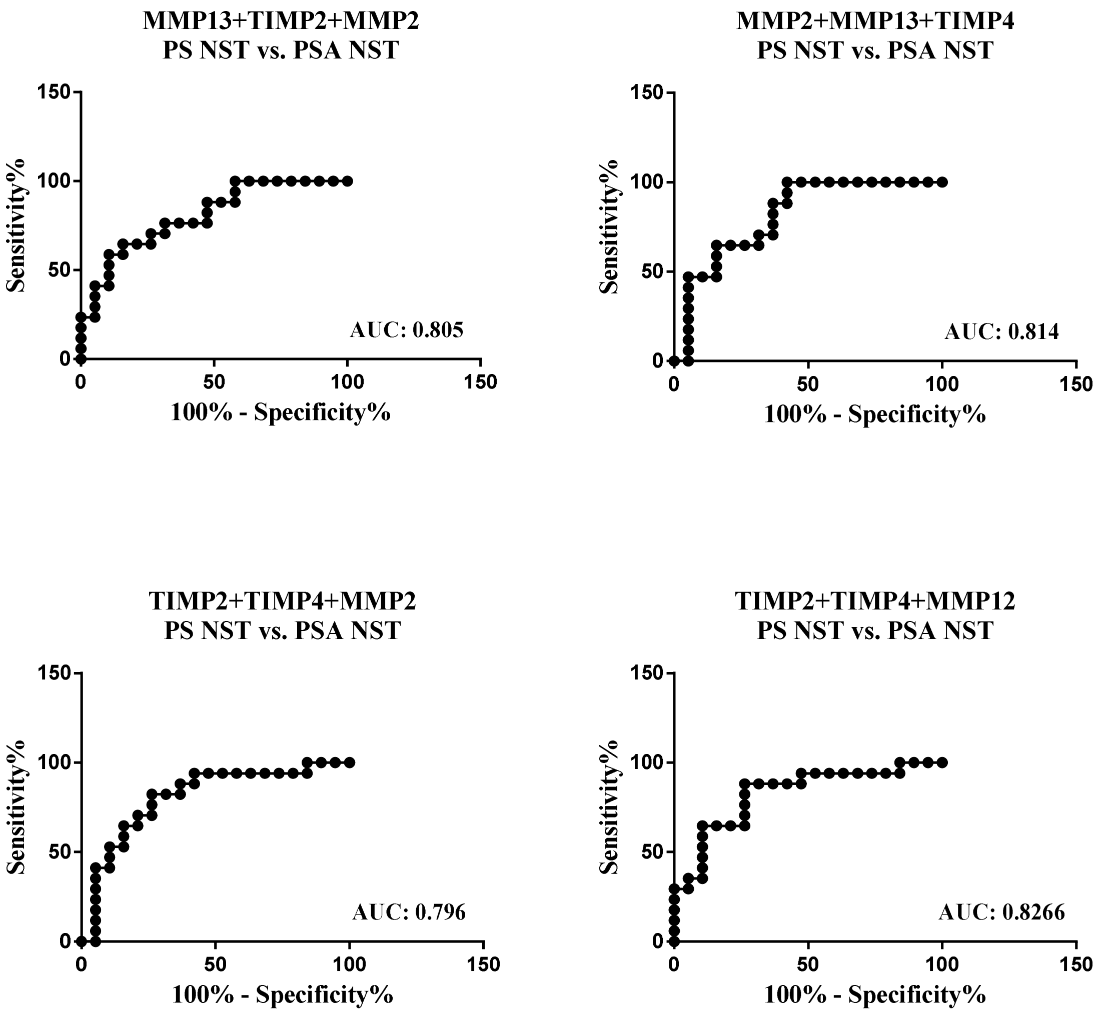
MMP13+TIMP2+MMP2
PS NST vs. PSA NST
AUC: 0.805
MMP2+MMP13+TIMP4
PS NST vs. PSA NST
AUC: 0.814
TIMP2+TIMP4+MMP2
PS NST vs. PSA NST
AUC: 0.796
TIMP2+TIMP4+MMP12
PS NST vs. PSA NST
AUC: 0.8266
Sensitivity%
100% - Specificity%
0
50
100
150

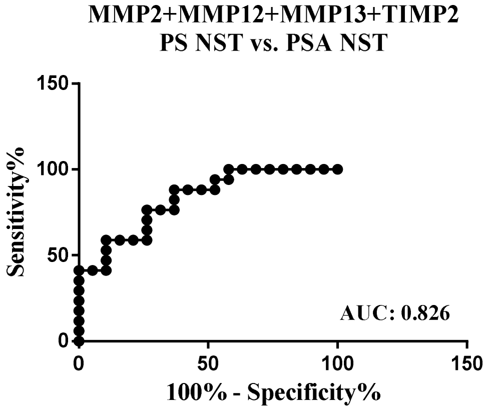
MMP2+MMP12+MMP13+TIMP2
PS NST vs. PSA NST
Sensitivity%
150
100
50
0
AUC: 0.826
0
50
100
150
100% - Specificity%

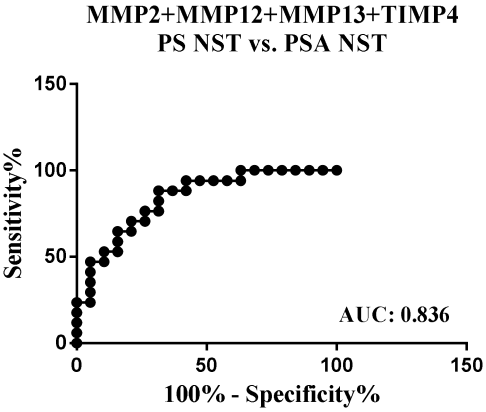
MMP2+MMP12+MMP13+TIMP4
PS NST vs. PSA NST
Sensitivity%
150
100
50
0
AUC: 0.836
0
50
100
150
100% - Specificity%

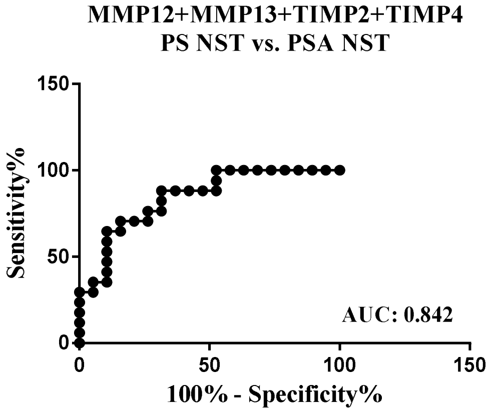
MMP12+MMP13+TIMP2+TIMP4
PS NST vs. PSA NST
Sensitivity%
150
100
50
0
AUC: 0.842
0
50
100
150
100% - Specificity%

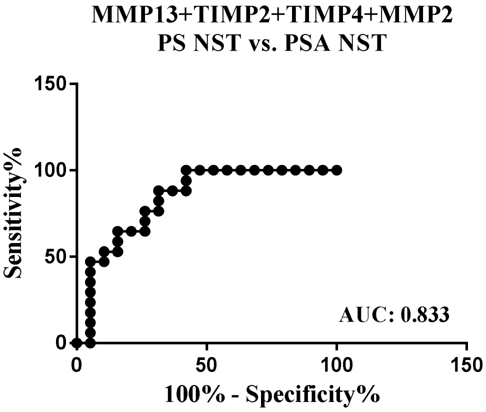
MMP13+TIMP2+TIMP4+MMP2
PS NST vs. PSA NST
Sensitivity%
150
100
50
0
AUC: 0.833
0
50
100
150
100% - Specificity%

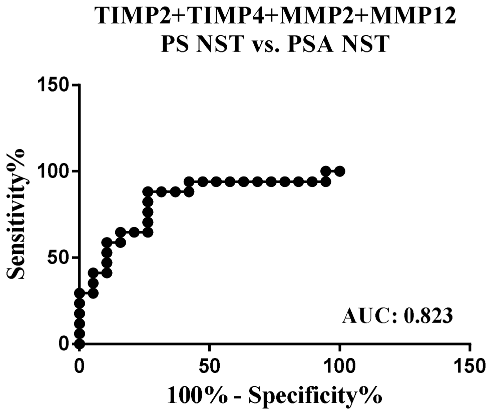
TIMP2+TIMP4+MMP2+MMP12
PS NST vs. PSA NST
Sensitivity%
150
100
50
0
AUC: 0.823
0
50
100
150
100% - Specificity%

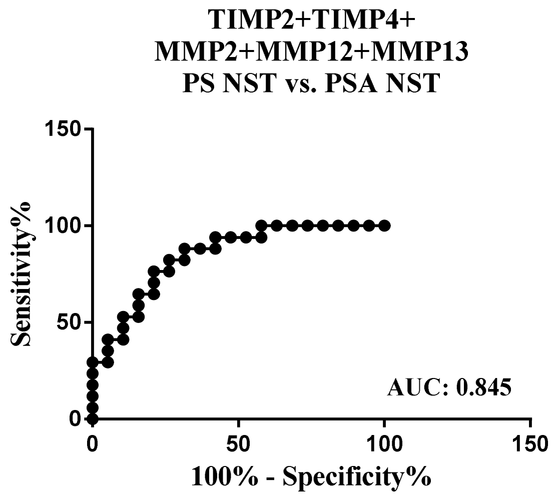
TIMP2+TIMP4+
MMP2+MMP12+MMP13
PS NST vs. PSA NST
150
100
50
0
Sensitivity%
AUC: 0.845
0
50
100
150
100% - Specificity%

Figure S2: ROC Curve Analysis of both single markers and their combinations in patients not systemically treated.

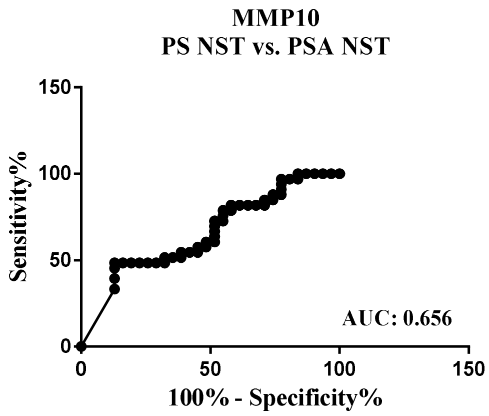

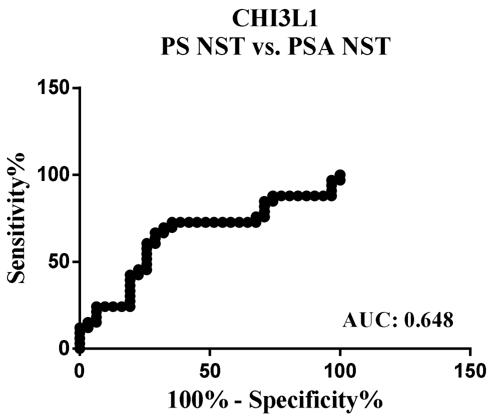

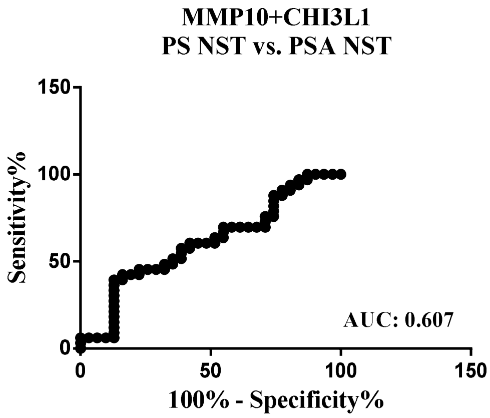

Figure S3: ROC Curve Analysis of MMP8 in patients with onychopathy.

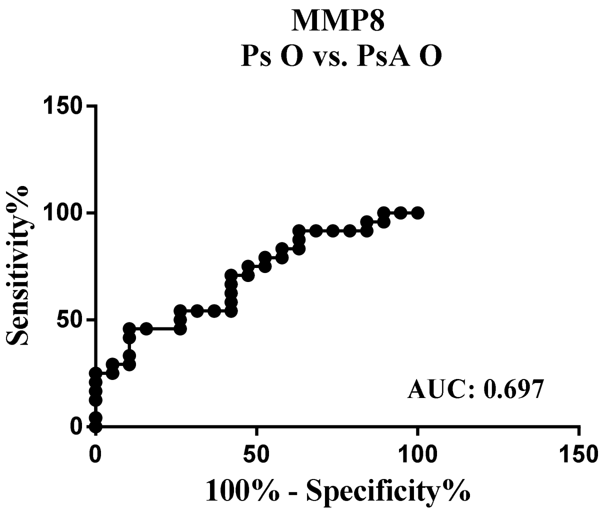

Supplement: Supplementary file 1 [file ijms-20-05617-s001.pdf]
